# Genotypic Variation in Nitrogen Utilization Efficiency of Oilseed Rape (*Brassica napus*) Under Contrasting N Supply in Pot and Field Experiments

**DOI:** 10.3389/fpls.2017.01825

**Published:** 2017-10-27

**Authors:** Huiying He, Rui Yang, Yajun Li, Aisheng Ma, Lanqin Cao, Xiaoming Wu, Biyun Chen, Hui Tian, Yajun Gao

**Affiliations:** ^1^College of Natural Resource and Environment, Northwest A and F University, Xianyang Shi, China; ^2^Institute of Oil Crop Research, Chinese Academy of Agricultural Sciences, Wuhan, China; ^3^Key Laboratory of Plant Nutrition and the Agri-environment in Northwest China, Ministry of Agriculture, Yangling, China

**Keywords:** canola, nitrogen use efficiency, variety trials, seeds per silique, nitrogen harvest index, harvest index

## Abstract

Oilseed rape (*Brassica napus*) characteristically has high N uptake efficiency and low N utilization efficiency (NUtE, seed yield/shoot N accumulation). Determining the NUtE phenotype of various genotypes in different growth conditions is a way of finding target traits to improve oilseed rape NUtE. The aim of this study was to compare oilseed rape genotypes grown on contrasting N supply rates in pot and field experiments to investigate the genotypic variations of NUtE and to identify indicators of N efficient genotypes. For 50 oilseed rape genotypes, NUtE, dry matter and N partitioning, morphological characteristics, and the yield components were investigated under high and low N supplies in a greenhouse pot experiment and a field trial. Although the genotype rankings of NUtE were different between the pot experiment and the field trial, some genotypes performed consistently in both two environments. N-responder, N-nonresponder, N-efficient and N-inefficient genotypes were identified from these genotypes with consistent NUtE. The correlations between the pot experiment and the field trial in NUtE were only 0.34 at high N supplies and no significant correlations were found at low N supplies. However, Pearson coefficient correlation (r) and principal component analysis showed NUtE had similar genetic correlations with other traits across the pot and field experiment. Among the yield components, only seeds per silique showed strong and positive correlations with NUtE under varying N supply in both experiments (*r* = 0.47^**^; 0.49^**^; 0.47^**^; 0.54^**^). At high and low N supply, NUtE was positively correlated with seed yield (*r* = 0.45^**^; 0.53^**^; 0.39^**^; 0.87^**^), nitrogen harvest index (NHI, *r* = 0.68^**^; 0.82^**^; 0.99^**^; 0.89^**^), and harvest index (HI, *r* = 0.79^**^; 0.83^**^; 0.90^**^; 0.78^**^) and negatively correlated with biomass distribution to stem and leaf (*r* = −0.34^**^; −0.45^**^; −0.37^**^; 0.62^**^), all aboveground plant section N concentration (*r* from −0.30^*^ to −0.80^**^), N distribution to the vegetative parts (silique husk, stem and leaf) (*r* from −0.40^**^ to −0.83^**^). N-efficient (N-responder) genotypes produced more seeds per silique and had significantly higher NHI and HI than did N-inefficient (N-nonresponder) genotypes. In conclusion, across the pot and field experiments, the 50 genotypes had similar underlying traits correlated with NUtE and seeds per silique may be a good indicator of NUtE.

## Introduction

Rapeseed (*Brassica napus*) is one of the most important oilseed crops in the world. Nitrogen (N) is of great importance in cultivating oilseed rape because this species requires more N to produce one unit of yield than most arable crops, such as sugar beet (*Beta vulgaris* L.) and winter wheat (*Triticum aestivum* L.) (Sylvester-Bradley and Kindred, 2009). To produce 0.1 t of seeds, winter oilseed rape accumulates approximately 6 kg of N in Europe (Rathke et al., 2006) and 4.3–4.8 kg of N in China (Li et al., 2017). Although higher N fertilizer application rates have greatly increased oilseed rape yields during the past century, the economic return per unit N fertilizer has decreased and waste in the environmental N budget had increased (Duffy, 2011). Thus, genotypes with an increased N use efficiency (grain production per unit of N available in the soil, NUE; Moll et al., 1982) are of commercial interest due to their high yield in low N-input agriculture and their low N wastage (Svečnjak and Rengel, 2006; Balint and Rengel, 2008; Chardon et al., 2012; Ulas et al., 2013).

According to Moll et al. (1982), NUE has two primary components: nitrogen uptake efficiency (plant N uptake/soil available N, NUpE) and nitrogen utilization efficiency (grain yield/total N uptake, NUtE). Genotypic variation in NUE, NUpE and NUtE occurs in rapeseed (Fageria et al., 2008; White and Brown, 2010; Miersch et al., 2016). Sylvester-Bradley and Kindred (2009) showed the lower NUE of oilseed rape relative to other crops was caused by lower NUtE rather than NUpE, because it has an early developed extensive root system (Kamh et al., 2005), and incomplete N remobilization from the vegetative parts into the seeds (Aufhammer et al., 1994; Schjoerring et al., 1995; Hocking et al., 1997; Wiesler et al., 2001). NUpE is associated with root growth and N uptake rates. Several strategies have been applied specifically at the root level to improve crop NUE (Garnett et al., 2009; Den Herder et al., 2010; Shi et al., 2012; Smith and De Smet, 2012). However, improving NUtE is a much more complex task. There are many studies involving gene engineering to improve the plant processes of C/N storage and metabolism, signaling and regulation of N metabolism and translocation, remobilization and senescence, which are related to NUtE (Chardon et al., 2012; Mcallister et al., 2012; reviewed in Good et al., 2004; Xu et al., 2012). Detailed investigations to identify the underlying traits of NUtE under various fertilization supplies are required to pinpoint the critical factors of N utilization within plant and maximal oilseed rape production. Nyikako et al. (2014) reported that a shorter oilseed rape plant might exhibit higher NUtE than taller ideotypes. Schulte auf'm Erley et al. (2011) found that N utilization and harvest index contributed to N efficiency depending on N supply. Furthermore, Berry et al. (2010) and Ulas et al. (2013) showed that N uptake post-flowering was an important trait underlying genotypic variation in yield at low and high N supply.

Seed yield of oilseed rape is highly variable (Rondanini et al., 2012). It is a complex trait that directly results from a combination of single-seed weight, seeds number per silique and siliques number per plant (Bouchet et al., 2016). It is also indirectly influenced by plant height, first valid branch height, number of first valid branches, and plant biomass (Zhao et al., 2016). Genotypic variation in these traits exists in oilseed rape (Diepenbrock, 2000; Wang et al., 2011; Chen et al., 2014) and is influenced by N supply (Bouchet et al., 2014; Ma et al., 2015). Significant correlations between seed yield and NUtE in oilseed rape exist (Berry et al., 2010; Erley et al., 2011; Yang et al., 2012; Ulas et al., 2013; Nyikako et al., 2014). Nonetheless, there is a lack of knowledge pinpointing the relationship between plant N utilization and these yield related traits, this information is needed to improve NUE in oilseed rape.

The long-term goal of improving the NUE of oilseed rape is to use biotechnology and crop breeding with best N management in the field (Diepenbrock, 2000; Bouchet et al., 2016). It is necessary to test plant phenotypes under field conditions because this testing procedure shows genotype performance in the target environment. It is also of great importance to evaluate genotypes in controlled environments, such as pots and hydroponic growth chambers. These basic tools reduce the complexity of environmental variation, making it possible for researchers to characterize plant at both root and shoot level (Den Herder et al., 2010) and use many methods (transcriptomic, metabolomic, and proteomic analyses) to evaluate NUE phenotypes and study NUE-related target genes (Mcallister et al., 2012). When comparing pot data to field data, it is important to compare phenotypes of identical genotypes from simplistic to complex growth conditions (Beatty et al., 2010). Although, there were several studies comparing phenotypes from controlled and field conditions (Cornelissen et al., 2003; Berry et al., 2010; Limpens et al., 2012; Poorter et al., 2016; Kohler, 2002), few studies have focused on NUE phenotypes of the same oilseed rape cultivars under both pot and field conditions (Hohmann et al., 2016).

In the present study, 50 oilseed rape cultivars were grown in pot and field experiments. This study was aimed at comparing the NUtE components of a variety of oilseed rape genotypes, grown under contrasting N application rates from simplistic (pot) to complex (field) growth conditions and identifying the phenotypes of oilseed rape with high N utilization efficiency.

## Materials and methods

### Plant materials

The 50 oilseed rape (*Brassica napus* L.) genotypes that were studied were provided by the Oil Crops Research Institute of the Chinese Academy of Agricultural Sciences and for simplicity were numbered as No. 1–50 (Table 1). The lines originated from 9 countries and included 19 accessions bred in the 1970s−1980s, 18 accessions bred in the 1980s−1990s, 6 accessions bred in the 1990s−2000s, and 8 accessions bred in 2000s−2011. These accessions included 12 commercial cultivars and other cultivars that were research resources in the lab. There were 7 spring and 43 winter cultivars among the 50 accessions.

**Table 1:** Origin, ecotype and breeding period of oilseed rape genotype.

**No**.	**Genotype**	**Origin**	**Type^a^**	**Breeding date**	**No**.	**Genotype**	**Origin**	**Type^a^**	**Breeding date**	**No**.	**Genotype**	**Origin**	**Type^a^**	**Breeding date**
1	Sangaoyoucai	China	SW	1970s	18	85-110 (parent)	China	SW	1980s	35	Legenol	Sweden	S	1980s
2	106	China	W	1970s	19	85-222	China	SW	1980s	36	Bechyne3	France	W	1980s
3	820	China	SW	1970s	20	Qianyoudijie 1	China	SW	1980s	37	Jian 123	China	SW	1990s
4	Huayou 14	China	SW	1970s	21	Guiyou 3	China	SW	1980s	38	Jie 53	China	SW	1990s
5	Huangyou 1	China	SW	1970s	22	Wesbery-1	Australia	S	1980s	39	Zhongshuang 4	China	SW	1990s
6	6024-2	China	SW	1970s	23	NPZ73	Germany	W	1980s	40	Jian 72	China	SW	1990s
7	6020-1	China	SW	1970s	24	PF2886	Germany	W	1980s	41	H40(343)	Czech	W	1980s
8	77023	China	SW	1970s	25	2909	Germany	W	1980s	42	Zhongshuang 10	China	SW	2000s
9	71-8	China	SW	1970s	26	28960	Germany	W	1980s	43	Zhongshuang 7	China	SW	2000s
10	Xinyou 1	China	W	1970s	27	Bridger	Germany	W	1980s	44	Sollux	Germany	W	1970s
11	3-105	China	SW	1970s	28	Moneta	Canada	S	1980s	45	Zhongshuang 9	China	SW	2000s
12	131178	/	SW	1970s	29	Heateol	Canada	S	1980s	46	Xiwang 106	China	SW	2000s
13	Libra	Poland	W	1970s	30	GSB627	China	SW	1990s	47	Huyou 18	China	SW	2000s
14	Gonda	Canada	W	1970s	31	Peixuan 171	China	SW	2000s	48	Zheyou 18	China	SW	2000s
15	Topas-15	France	S	1970s	32	Huashuang 3	China	SW	2000s	49	Huangzi 2	China	W	1990s
16	H33	France	S	1970s	33	Peixuan 170	China	SW	1970s	50	H49	Russia	W	1970s
17	88 Jian 6	China	SW	1980s	34	Parter	Germany	W	1980s					

a *W, winter ecotype; SW, semi-winter ecotype; S, spring ecotype*.

In the pot experiment, locally collected red loess soil (Earth-cumuli-Orthic Anthrosols; 108°E, 34°15′N) was used with a pH of 7.7. The soil properties were as follows: organic matter 7.5 g kg^−1^, total N 0.72 g kg^−1^, available N 21.4 mg kg^−1^, total P 0.50 g kg^−1^, available P 7.6 mg kg^−1^ (Olsen P), total K 19.5 g kg^−1^, and available K 73.1 mg kg^−1^.In the field experiment, the soil was alluvial soil (Fluvo-aquic soils) with a pH of 7.9, organic matter 15.6 g kg^−1^, total N 1.0 g kg^−1^, available N 24.0 mg kg^−1^, total P 0.5 g kg^−1^, available P 12.2 mg kg^−1^, total K 22.0 g kg^−1^, and available K 168.0 mg kg^−1^. The available N in the pot and in the field experiments fell within the range defined as N deficient (Wang et al., 1985; Liu and Tu, 1989; Zou Juan, 2010).

### Experiments design

#### Pot experiment

In the pot experiment, 50 oilseed rape genotypes were grown in a glasshouse at the Northwest A and F University, Yangling (108°E, 34°15′N), Shaanxi province, China. There were two N fertilizer application rates, high N (0.3 g of N kg^−1^ dry soil) and low N (0.1 g of N kg^−1^ dry soil) level. N fertilizer was applied as urea. Other nutrients were applied in sufficient amounts: superphosphate (P_2_O_5_ 0.2 g kg^−1^ dry soil) and potassium chloride (K_2_O 0.3 g kg^−1^ dry soil). Borax (B 1.0 mg kg^−1^ dry soil) was applied with irrigation at the rosette stage. The Fe and Zn fertilizers were sprayed on the plant at the stem elongation stage and blossom stage. Each pot contained 9 kg of air-dried soil and the experiment was conducted in three replicates.

The seeds were hand sown on 1 Nov. 2007. Eight uniform seedlings per pot were maintained for a few days before the stem elongation stage. After that stage, only a single plant was retained per pot. All plants were harvested at physiological maturity. Early maturing genotypes were harvested on 18 May 2008, and late-maturing genotypes were harvested on 1 Jun. 2008. Throughout the experiment, each pot was regularly weighed to estimate water loss, and tap water was added to maintain the soil moisture at 60–75% of field capacity.

#### Field experiment

The field experiment was conducted at the Agri-experiment Station, Northwest A and F University, with an average annual rainfall of 649 mm and an average annual temperature of 13°C. Rainfall is mainly received from July to September. The total rainfall was 629.7 mm from 1 Sep. 2009 to 31 Aug. 2010. This was a typical year compared to the long-term mean rainfall, with 326.4 mm of rainfall during the field trial period (from September to May). The winter at Yangling is mild and winter and spring oilseed rapes grown simultaneously can survive through the winter and form normal seeds in the following year. The genotypes were arranged in a split-block design. The main treatment was the N fertilizer rate (high N, 180 kg N hm^−2^; low N, 0 kg N hm^−2^) with 4 replicates, and the sub-treatment was the rapeseed genotype with one-row-plot. Superphosphate (P_2_O_5_ 135 kg hm^−2^) was applied to all of the treatments as base fertilizer on 24 Sep. 2009. Seeds were sown on 25 Sep. in one-row plots that were 4 m long and 0.4 m wide. The spacing was 0.4 m between rows and 0.15 m between plants within rows. The population density was 10 plants per plot, and manual thinning was used to reduce the distance between plants to 0.4 m in each row at 50 days after sowing. Each plant was manually harvested on the date at which approximately 80% of siliques appeared light -yellow to avoid seed shattering. The plants were harvested on 10 May for early maturing genotypes and on 17 May for late-maturing genotypes.

### Measurements and data analysis

At harvest (BBCH 89; Lancashire et al., 1991) plant height and first valid branch height of each plant were measured, and the numbers of first valid branch and siliques per plant were counted in the pot experiment. Then, 1,000 seeds were counted and weighed. The average number of seeds per silique was calculated from the seed yield, the number of siliques per plant and the 1,000-seed weight. In the field trial, 6 plants out of the one-row plot were used to measure these traits for each genotype.

In the pot experiment, each plant was dissected to stem and leaves attached-to-stem, seed, silique husk and root at grain harvest. Tap roots were carefully collected by washing the soil. In the field experiment, the 6 plants out of the one-row-plot were divided into 3 fractions: seed, silique husk, and stem and leaves. The average dry weight and N concentration of each plant fraction were measured for each genotype. The N concentration was analyzed using the Kjeldahl method (Bao, 2000).

The N utilization efficiency (NUtE) was calculated as follows:

NUtE = seeddryweight÷shootNaccumulation (Moll et al., 1982).

Shoot N accumulation = seed N accumulation + silique husk N accumulation + stem and leaf N accumulation.

The genotypes were classified using the system proposed by Gerloff (1977) and Rengel and Graham (1995). For both the high-N and low-N treatments, the median values of NUtE (taken as the average of the values of genotypes ranked 25th and 26th) were calculated in both the pot and the field experiments. Comparison of the mean NUtE of each cultivar with the medium-efficiency interval (median ± four standard errors of the genotype effect) facilitated the selection of N-responder (genotypes with a NUtE value above the medium-efficiency interval at high N in both the pot and field experiments), N-nonresponder (genotypes with a NUtE value below the medium-efficiency interval at high N in both the pot and field experiments), N-efficient (genotypes with a NUtE value above the medium-efficiency interval at low N in both the pot and field experiments) and N-inefficient (genotypes with a NUtE value below the medium-efficiency interval at low N in both the pot and field experiments) genotypes.

Data sets for NUtE, biomass, biomass ratio, N concentration, N accumulation, N distribution, yield components and morphological traits were subjected to a two-way ANOVA (SAS v8.1, Cary, NC, USA), including “N treatment” (low N rates and high N rates), “Genotype” and their interactions in the model. The ANOVA model for the field experiment used a split-plot design and for the pot experiment we used a fully randomized plot design. In the ANOVA analysis, replicates were regarded as random effects and N treatment and genotype as fixed effects. Pearson coefficient of correlations between traits and NUtE were calculated using the Proc CORR procedure of SAS. Simple linear regression was conducted using the Proc REG procedure of SAS. A Student's *t*-test was employed to compare the means of genotypes, with the significance level set at *P* < 0.05. All of the figures were created using the SigmaPlot software (v10.0, SAS Institute, 2003). A principal component analysis was carried out to show the genetic correlation pattern. Correlation matrix were computed from the pairwise genetic correlation coefficients between traits. Eigenvectors were obtained by the decomposition of the correlation matrix and then they were standardized by multiplying them with the square root of the corresponding eigenvalue. SAS JMP10 was employed to carry out the principal component analysis.

## Results

### Genotypic variations of NUtE in oilseed rape in both pot and field experiments

**Figure 1:**
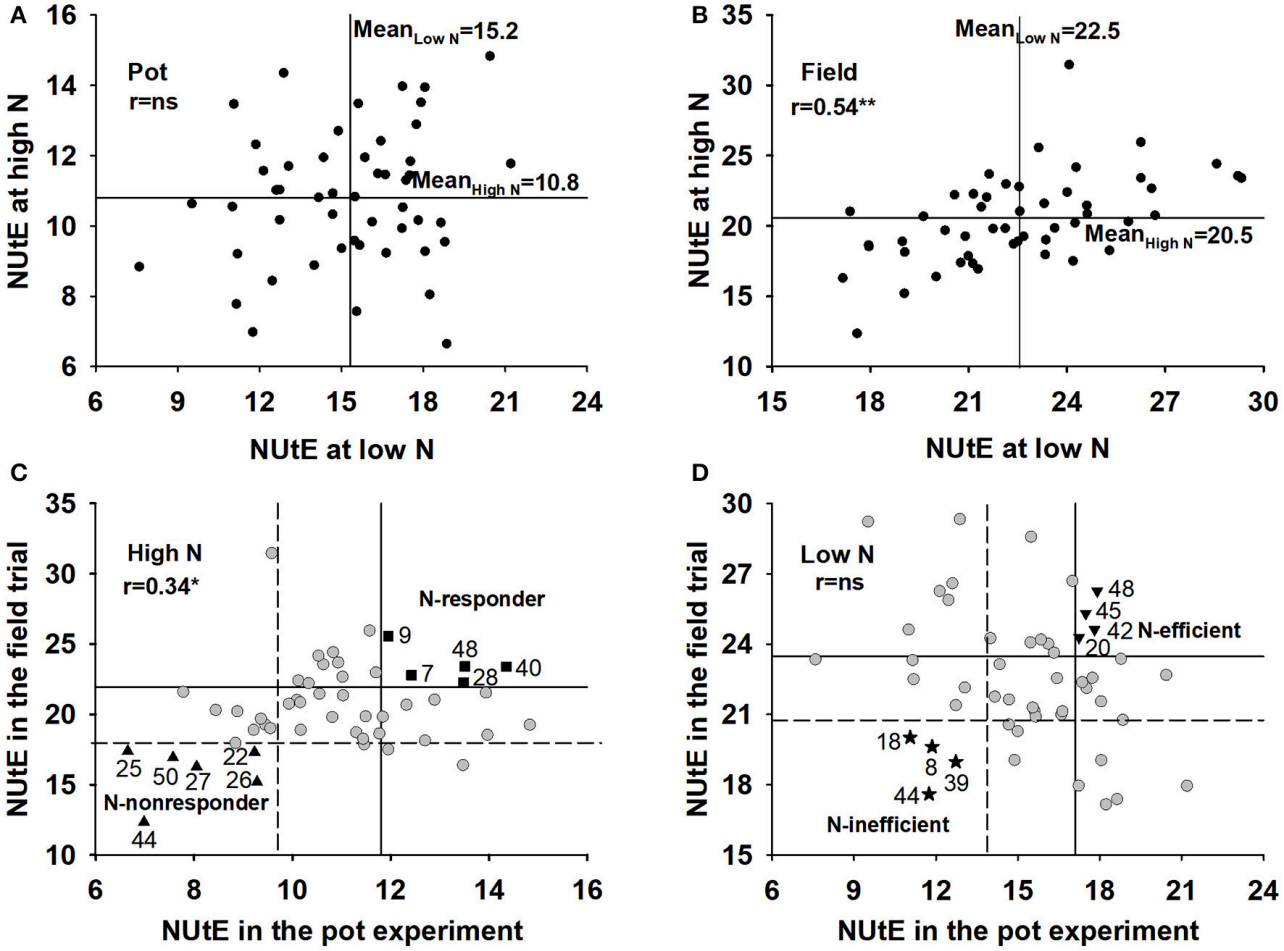
Mean NUtE for 50 oilseed rape genotypes under high and low N supply in a the pot experiment **(A)** and a the field experiment **(B)**, selection for N-responder and N-nonresponder genotypes at high N rates in the pot and field experiments **(C)** and N-efficient and N-inefficient genotypes at low N rates in the pot and field experiments **(D)**. NUtE intervals at four standard errors of the genotype effect above and below the median are indicated by vertical lines in the pot experiment **(C,D)** and by horizontal lines in the field experiment **(C,D)**.The horizontal line represents the mean of the genotypes in the pot experiment and the vertical line represents the mean in the field experiment **(A,B)**. r means Pearson Coefficient correlations between x axis and y axis. ns means not significant; ^*^means significant at *p* < 0.05.

The NUtE of 50 rapeseed genotypes was evaluated at grain harvest. The genotypes significantly differed in NUtE (Figures 1A,B; *p* < 0.001) and performed differently in terms of NUtE across the pot and field experiments (Supplementary Table 1). Under a low N supply, the average NUtE of the 50 genotypes was 15.2 kg seed yield kg^−1^ shoot N uptake in the pot experiment and 22.5 kg seed yield kg^−1^ shoot N uptake in the field experiment, whereas under a high N level, the average NUtE was 10.8 kg seed yield kg^−1^ shoot N uptake in the pot experiment and 20.5 kg seed yield kg^−1^ shoot N uptake in the field experiment.

According to the classification scheme of N efficiency and N response proposed by Gerloff (1977), these genotypes were distributed over all four quadrants, which allowed the discrimination of efficient, inefficient, responder and non-responder genotypes and facilitated the selection of underlying traits for NUtE. Under both the high and low N supplies, the rankings of genotypes in NUtE were different between the pot experiment and field trial, which means most genotypes performed differently and show a huge interaction with the cultivation environment. However, some genotypes performed consistently at both the controlled (pot experiment) and the complex (field trial) growth conditions. Based on their NUtE values, 5 genotypes were ranked as N-responder, 6 genotypes were ranked as N-nonresponder, 4 genotypes were ranked as N-efficient and 4 genotypes were ranked as N-inefficient in the pot and field experiments (Figures 1C,D). Most N-responder genotypes and N-efficient genotypes were not the same except genotype 48 which is both N-responding and N-efficient. Under both the pot and field experiments, N-responders had significantly higher NUtE values than did N-nonresponders under high N supplies (*p* < 0.0001) and N-efficient genotypes had significantly higher NUtE values than did the N-inefficient genotypes under low N supplies (*p* < 0.0001) (data not shown).

### Correlations of NUtE with yield components and shoot N accumulation

**Figure 2:**
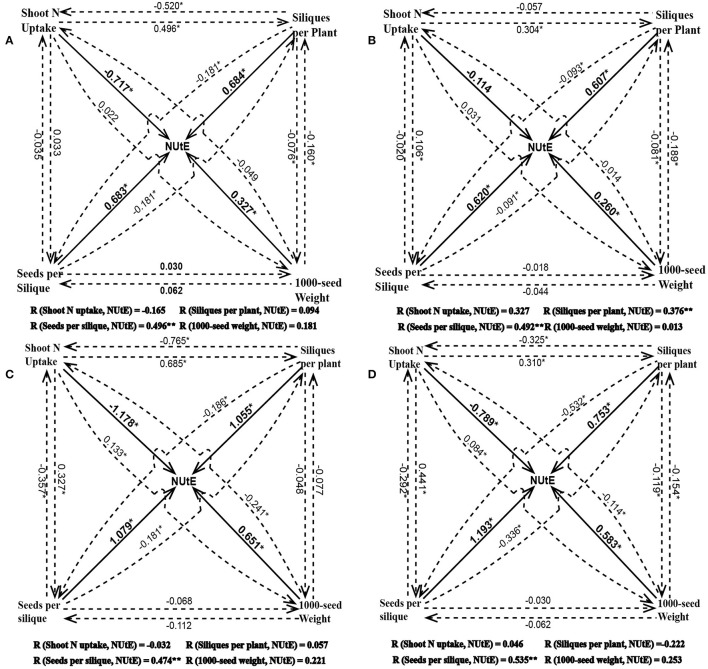
The Path coefficient for NUtE, yield components and shoot N uptake at high N treatment in the pot experiment **(A)**, at low N treatment in the pot experiment **(B)**, at high N treatment in the field trial **(C)**, and at low N treatment in the field trial **(D)**. The solid line represents the direct effect of a given parameter on NUtE and the dotted line represents the indirect effect of the corresponding parameter on NUtE via the other parameters. The value near a vector means effect of trait at the starting point on the trait at the terminal point of the vector (i.e., in A 0.683 is the direct effect value of seeds per silique on NUtE and −0.181 is the indirect effect value of seeds per silique on NUtE through siliques per plant). R is the Pearson coefficient of correlation (the final effect, i.e., 0.496 represents the sum of direct effect of seeds per silique on NUtE and the indirect effect of seeds per silique on NUtE through shoot N uptake, siliques per plant and 1000-seed weight). ^*^represents *p* < 0.05.

**Figure 3:**
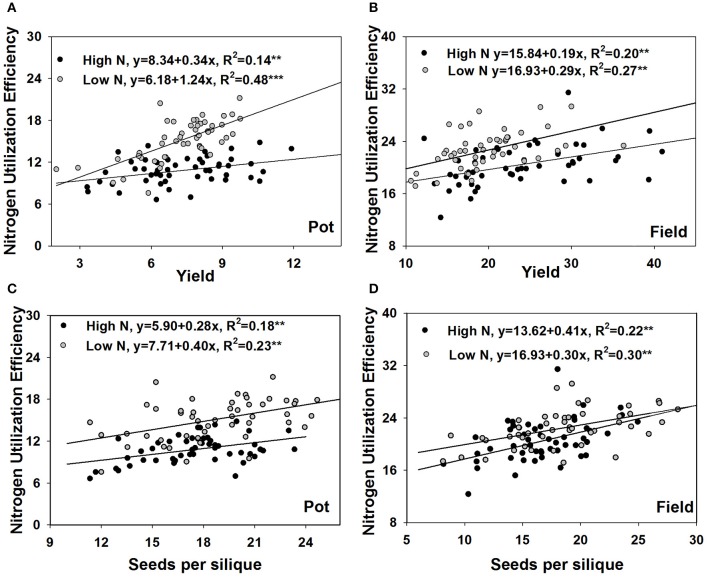
Regression analysis of NUtE with yield in the pot experiment **(A)** and the field experiment **(B)**, with seeds number per silique in the pot experiment **(C)** and the field experiment **(D)**.

NUtE could be divided into seed yield and shoot N accumulation. Seed yield has three components which are siliques per plant, seeds per silique and 1,000-seed weight. Under both the pot and field experiments, NUtE was significantly correlated with grain yield (*p* < 0.01), but not correlated with shoot N accumulation (Figure 2). Among the three yield component characters, seed number per silique always had a strong positive correlation with NUtE under both the pot experiment and field trial while neither siliques number per plant nor 1,000-seed weight had a correlation with NUtE under all of the growth conditions (Figure 2). The strong correlations between NUtE and yield, seed number per silique were also confirmed by linear regression analysis. Every single gram increase of yield was associated an increase in NUtE of 0.19 to 1.24 kg seed yield kg^−1^ shoot N uptake, which depended on the growth conditions (Figures 3A,B). Every one seed increase per silique was associated with an increase in NUtE of 0.28–0.41 kg seed yield kg^−1^ shoot N uptake (Figures 3C,D).

### Correlations between NUtE and HI, NHI

**Figure 4:**
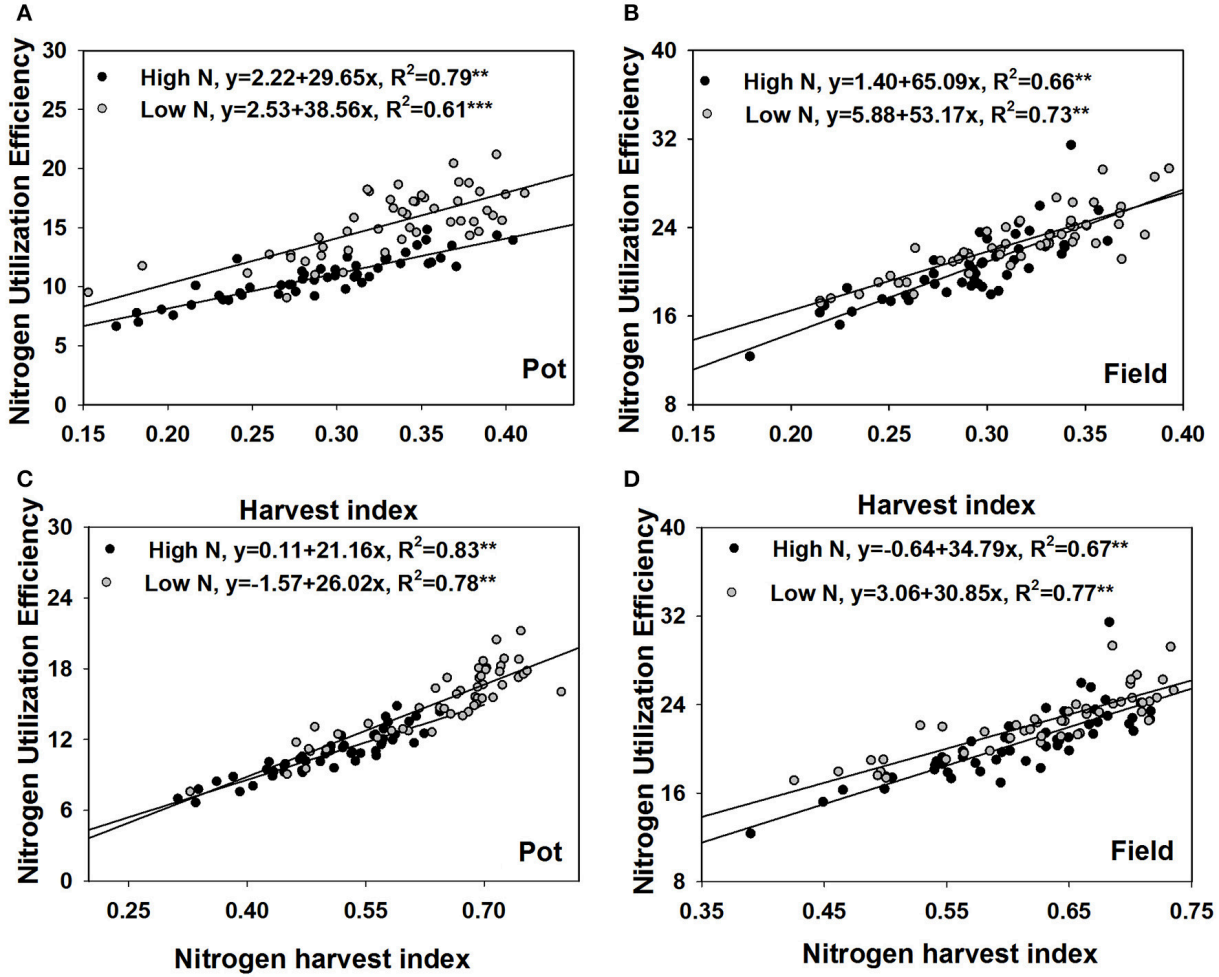
Regression analysis of NUtE with harvest index in the pot experiment **(A)** and the field experiment **(B)**, with nitrogen harvest index in the pot experiment **(C)** and the field experiment **(D)**.

Under both contrasting N treatments, only grain yield was always significantly correlated with NUtE (*p* < 0.01 Table 2) in both the pot experiment and the field trial. Under high N supplies, the biomasses of stem and leaf, silique husk and root were significantly correlated with NUtE in the pot experiment (*p* < 0.05). By contrast in the field trial, no similar biomass and NUtE correlations were found. NUtE was positively correlated with the ratios of grain to shoot biomass (i.e., harvest index, HI) (*p* < 0.01) and negatively correlated with the ratios of stem and leaf to shoot biomass (*p* < 0.01) (Table 2) in both the pot and field experiments. Under high N treatments, no significant correlation was found between NUtE and the ratio of silique husk to shoot biomass whereas NUtE was negatively correlated with the ratio of silique husk to shoot biomass under the low N treatments (*p* < 0.01). Linear regression analysis showed that under high N treatment every 0.01 increase of harvest index was associated with an increase in NUtE of 0.30 kg seed yield kg^−1^ shoot N uptake in the pot experiment and an increase of 0.65 kg seed yield kg^−1^ shoot N uptake in the field trial (Figures 4A,B). At low N treatments, every 0.01 increase of harvest index was associated with an increase in NUtE of 0.39 kg seed yield kg^−1^ shoot N uptake in the pot experiment and an increase of 0.53 kg seed yield kg^−1^ shoot N uptake in the field experiment.

**Table 2:** Correlations between NUtE and biomass characteristics at high-N and low-N application rates and analysis of variance results of biomass characteristics in the pot and field experiments (*n* = 50).

**N level**	**Correlations with NUtE**				**ANOVA**					
	**Field experiment**		**Pot experiment**		**Field experiment**			**Pot experiment**		
	**High N**	**Low N**	**High N**	**Low N**	**G**	**N**	**G × N**	**G**	**N**	**G × N**
Grain yield (g plant^−1^)	0.45^**^	0.53^**^	0.39^**^	0.87^**^	^***^	^*^	NS	^***^	^*^	NS
Silique husk biomass (g plant^−1^)	0.03^NS^	−0.21^NS^	−0.28^*^	0.01^NS^	^***^	NS	NS	^***^	^***^	NS
Stem and leaf biomass (g plant^−1^)	−0.09^NS^	−0.08^NS^	−0.47^**^	−0.03^NS^	^***^	^*^	NS	^***^	^***^	^**^
Shoot biomass (g plant^−1^)	0.13^NS^	0.12^NS^	−0.28^*^	0.32^*^	^***^	^*^	NS	^***^	^***^	^*^
Root biomass (g plant^−1^)			−0.33^*^	0.21^NS^				^***^	^***^	NS
Whole plant biomass (g plant^−1^)			−0.31^*^	0.31^*^				^***^	^***^	^*^
Grain biomass/Shoot biomass	0.79^**^	0.83^**^	0.90^**^	0.78^**^	^***^	^*^	NS	^***^	^***^	^***^
Silique husk biomass/Shoot biomass	−0.23	−0.67^**^	0.01	−0.44^**^	^***^	NS	NS	^***^	NS	NS
Stem and leaf biomass/Shoot biomass	−0.45^**^	−0.37^**^	−0.62^^**^^	−0.34^*^	^***^	NS	NS	^***^	^***^	^**^
Root biomass/Whole plant biomass			−0.30^*^	0.19^NS^				^***^	NS	NS

NS, not significant;

* significant at p ≤ 0.05;

** significant at p ≤ 0.01;

*** *significant at p ≤ 0.001. G, genotype*.

Under both N supply rates, NUtE was negatively correlated with grain N concentration, stem and leaf N concentration and silique husk N concentration in both the pot and field experiments (*p* < 0.05, Table 3). However, no significant correlation was found between NUtE and root N concentration in the pot experiment (Table 3). Under high N treatment, NUtE was not correlated with grain N accumulation. In contrast, significantly positive correlations were found between NUtE and grain N under the low N treatment in both the pot and field experiments (*p* < 0.05 or *p* < 0.01). As for the stem and leaf N and silique husk N accumulations, they showed negative correlations with NUtE under contrasting N rates in both the pot and field experiments (Table 3). No significant correlation was found between NUtE and root N accumulation in the pot experiment. While the ratios of stem and leaf N accumulation to shoot N and the ratio of silique husk N accumulation to shoot N accumulation were negatively correlated with NUtE, the ratio of grain N accumulation to shoot N accumulation (i.e., N harvest index, NHI) was positively correlated with NUtE. Linear regression analysis showed that every 0.01 increase of NHI was associated with an increase in NUtE of 0.21–0.34 kg seed yield kg^−1^ shoot N uptake (Figures 4C,D). The differences among the extreme genotypes in NUtE were consistent with the correlations between N traits and NUtE (Supplementary Figures 1–3).

**Table 3:** Correlations between NUtE and N traits at high-N and low-N application rates and analysis of variance results of N traits in the field and pot experiments (*n* = 50).

**N level**	**Correlations with NUtE**				**ANOVA results**					
	**Field trial**		**Pot experiment**		**Field trial**			**Pot experiment**		
	**High N**	**Low N**	**High N**	**Low N**	**G**	**N**	**G × N**	**G**	**N**	**G × N**
Grain N concentration (g kg^−1^)	−0.55^**^	−0.30^*^	−0.35^*^	−0.66^**^	NS	NS	NS	^***^	^***^	^***^
Silique husk N concentration (g kg^−1^)	−0.71^**^	−0.40^**^	−0.54^**^	−0.80^**^	^*^	NS	NS	^***^	^***^	^***^
Stem and leaf N concentration (g kg^−1^)	−0.75^**^	−0.72^**^	−0.30^*^	−0.47^**^	^***^	NS	NS	^***^	^***^	^***^
Root N concentration (g kg^−1^)			0.01^NS^	−0.06^NS^				^***^	^***^	NS
Grain N accumulation (g plant^−1^)	0.27	0.34^*^	0.27^NS^	0.63^**^	^***^	^*^	NS	^***^	^***^	^**^
Silique husk N accumulation (g plant^−1^)	−0.28^*^	−0.35^*^	−0.44^**^	−0.61^**^	^*^	NS	NS	^***^	^***^	^***^
Stem and leaf N accumulation (g plant^−1^)	−0.52^**^	−0.48^**^	−0.48^**^	−0.33^*^	^***^	NS	NS	^***^	^***^	^***^
Shoot N accumulation (g plant^−1^)	0.01^NS^	0.04^NS^	−0.09^NS^	0.32^*^	^***^	^*^	^*^	^***^	^***^	^***^
Root N accumulation (g plant^−1^)			0.05^NS^	0.20^NS^				^***^	^***^	NS
Plant N accumulation (g plant^−1^)			−0.27^NS^	0.34^*^				^***^	^***^	^***^
Grain N/shoot N	0.68^**^	0.82^**^	0.99^**^	0.89^**^	^***^	NS	NS	^***^	^***^	^***^
Silique husk N/shoot N	−0.44^**^	−0.72^**^	−0.40^**^	−0.83^**^	^***^	NS	NS	^***^	^***^	NS
Stem and leaf N/shoot N	−0.70^**^	−0.63^**^	−0.56^**^	−0.56^**^	^***^	NS	NS	^***^	^***^	^***^
Root N/Plant N			−0.28^*^	0.16^NS^				^***^	^**^	^**^

NS, not significant;

* significant at p ≤ 0.05;

** significant at p ≤ 0.01;

*** *significant at p ≤ 0.001. G, genotype*.

**Figure 5:**
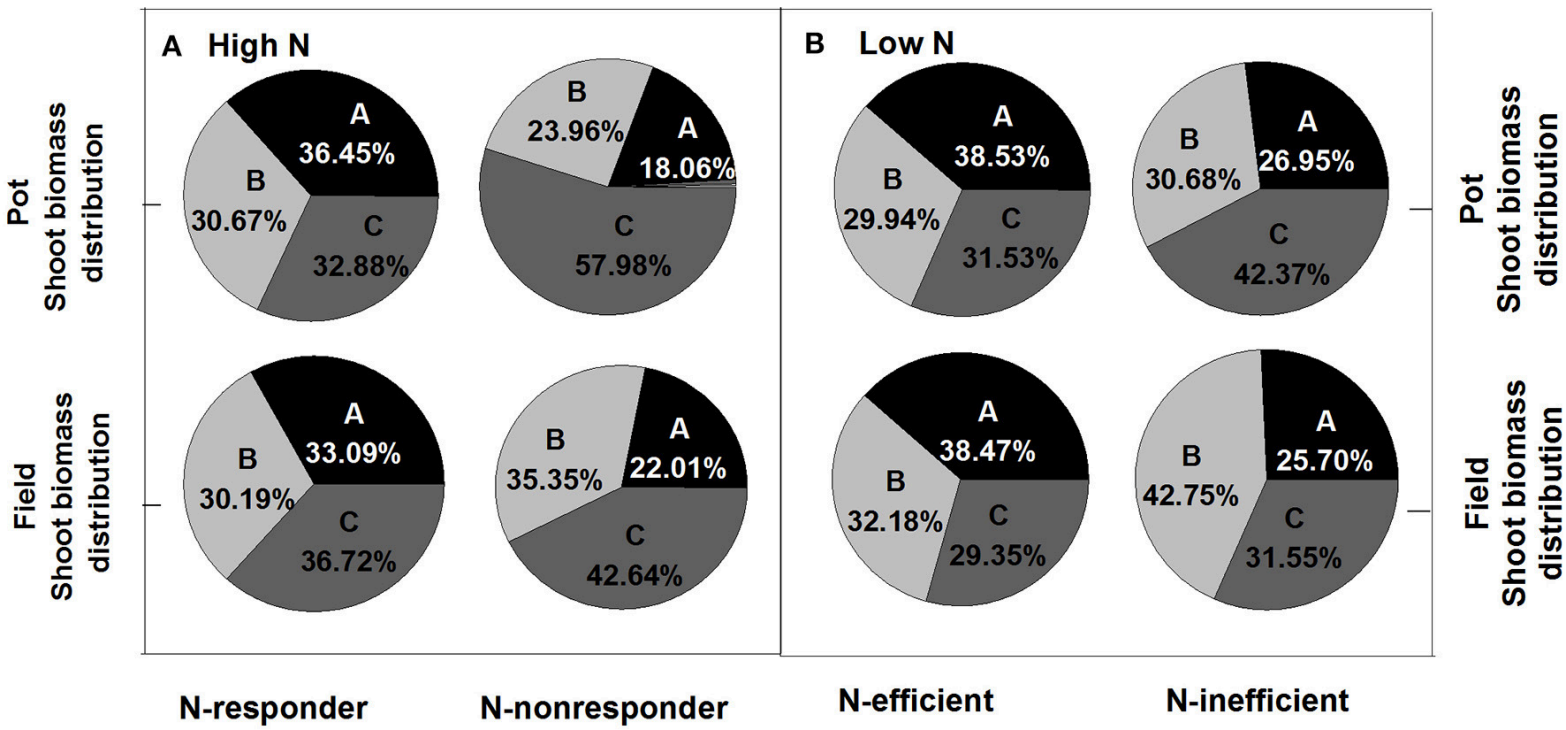
Ratios of plant section to shoot biomass of N-responder and N-nonresponder genotypes under high N rates **(A)** and N-efficient and N-inefficient genotypes under low N rates **(B)**. A, grain; B, silique husk; C, stem and leaf.

**Figure 6:**
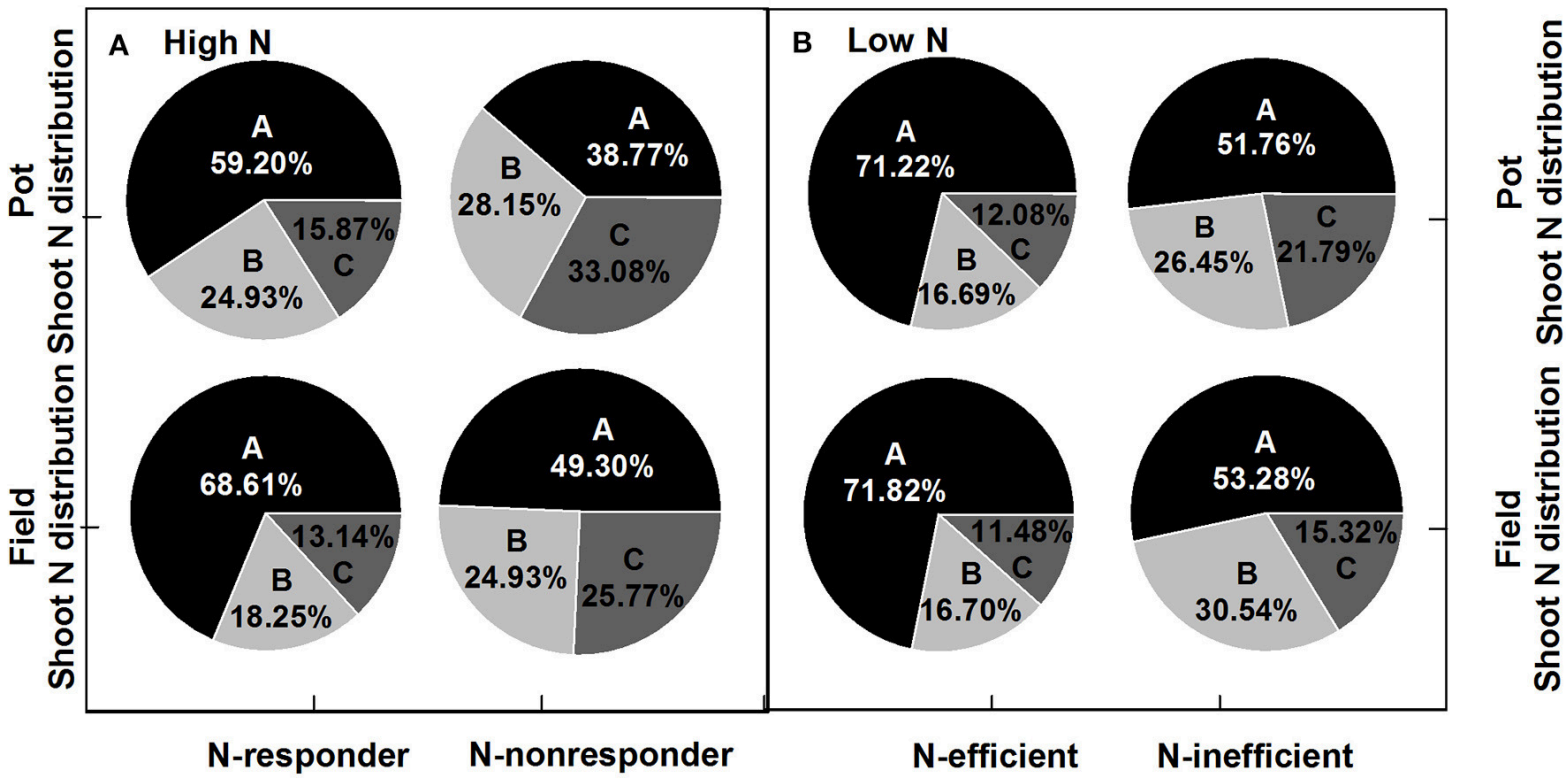
N uptake ratios of plant section to shoot of N-responder and N-nonresponder genotypes at high N rates **(A)** and of N-efficient and N-inefficient genotypes at low N rates **(B)**. A, grain; B, silique husk; C, stem and leaf.

Under both high and low N supplies, the high N efficiency genotypes (N-responder at high N supply or N-efficient genotype at low N supply) tended to have higher seed yield and more biomass to the seed than the low N efficiency genotypes (N-nonresponder at high N supply or N-inefficient genotype at low N supply) (Figure 5; Supplementary Figure 1). Under high N supplies, the HI of N-responders in the pot experiment was 36.45% while that of N-nonresponders was only 18.06% and in the field trial the HI of N-responders was 33.09% while those of N-nonresponders were 22.01%. Under low N supplies, the HI of N-efficient genotypes was about 38% in both the pot and field experiments while the HI of N-inefficient genotypes was about 26% in both two experiments. For plant N distribution, under high and low N supplies, the high N efficiency genotypes (N-responders at high N supply or N-efficient genotypes at low N supply) significantly had a high NHI than the low N efficiency genotypes (N-nonresponder at high N supply or N-inefficient genotype at low N supply) (Figure 6; *p* < 0.05) and the ratios of silique husk N and stem and leaf N to shoot N were significantly higher in the N-nonresponders and the N-inefficient genotypes than those in the N-responders and N-efficient genotypes in both the pot and field experiments (*p* < 0.05).

For the root samples, under high N supply, both the ratio of root biomass to whole plant biomass and the ratios of root N accumulation to whole plant N accumulation were negatively correlated with NUtE (*p* < 0.05) while at low N supply, no significant correlations were found between these two ratios and NUtE (Table 2). The N-responders had significantly lower ratios of root biomass to whole plant biomass (average 8.88%) than the N-nonresponders (average 15.28%) (*p* < 0.05). Those of the N-efficient genotypes (average 8.91%) also trended to be lower than N-inefficient genotypes (average 10.83%). Like the biomass data, the N-responders also distributed less N to the root (ratios of root N to whole plant N averaged 5.22%) relative to the N-nonresponders (ratios of root N to whole plant N averaged 9.15%).

### Correlations between NUtE and morphological traits

Though genotypes differed significantly in agronomic traits, no consistent correlations were found among morphological traits and NUtE in all of the growth conditions (Table 4). In the pot experiment, plant height, first valid branch height and the number of first valid branches were all significantly correlated with NUtE at high N treatment (*p* < 0.05), while at low N treatment, no significant correlations were found. However, in the field trial, only plant height and first valid branch height correlated with NUtE at low N treatment. At high N treatment, none of the three morphological traits correlated with NUtE. No significant differences in morphological traits were found between high N efficiency genotypes and low N efficiency genotypes (data not shown).

**Table 4:** Correlations between NUtE and morphological traits at high N and low N application rates and analysis of variance (ANOVA) results of morphological traits in the field and pot experiments (*n* = 50).

**N level**	**Correlations with NUtE**				**ANOVA**					
	**Field trial**		**Pot experiment**		**Field trial**			**Pot experiment**		
	**High N**	**Low N**	**High N**	**Low N**	**G**	**N**	**G × N**	**G**	**N**	**G × N**
Plant height (cm)	−0.31^*^	−0.22^NS^	−0.15^NS^	0.36^**^	^***^	^*^	NS	^***^	^**^	NS
First valid branch height (cm)	−0.46^**^	−0.25^NS^	−0.18^NS^	0.37^**^	^***^	^*^	NS	^***^	NS	NS
No. of first valid branches	0.59^**^	0.24^NS^	0.17^*NS*^	0.16^NS^	^***^	NS	NS	^***^	^**^	^*^

NS, not significant;

* significant at p ≤ 0.05;

** significant at p ≤ 0.01;

*** *significant at p ≤ 0.001. G, genotype*.

### Principal component analysis

**Figure 7:**
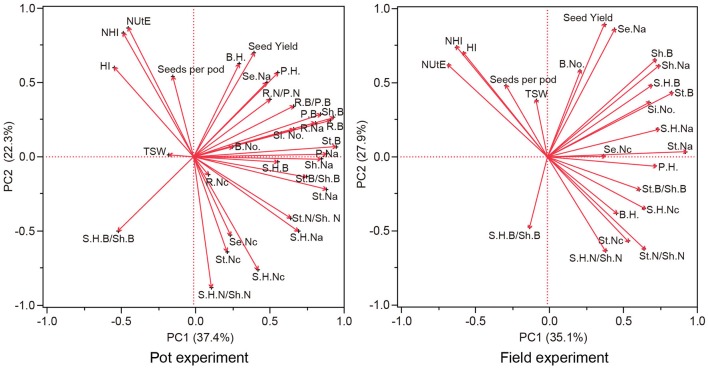
Component pattern for principal components 1 and 2 for genetic correlations of NUtE, seed yield and components, biomass distribution, N distribution and morphological traits. NUtE, nitrogen utilization efficiency; S.H.B., silique husk biomass; St.B., stem biomass; Sh.B., shoot biomass; R.B., root biomass; P.B., plant biomass; S.H.B/Sh.B, silique husk biomass/shoot biomass; St.B/Sh.B, stem biomass/plant biomass; P.H., plant height; B.H., branch height; B.No., branch number; Si.No., silique number; TSW, 1000-seed weight; Se.Nc., seed N concentration; S.H.Nc, silique husk N concentration; St.Nc, stem N concentration; R.Nc, root N concentration; Se.Na, seed N accumulation; S.H.Na, silique husk N accumulation; St.Na, stem N accumulation; Sh.Na, shoot N accumulation, R.Na, root N accumulation; P.Na, plant N accumulation; HI, harvest index; NHI, nitrogen harvest index; S.H.N/Sh.N, silique husk N/shoot N; St.N/Sh.N, stem N/shoot N.

The association between traits represented by the first and second principle component is depicted in Figure 7, and is similar between the pot and field experiments. Both principal components explained about 60% of the total variation. The first principal component (x-axis) is mainly spanned by traits of biomass, N concentration, N accumulation, ratios of biomass distributed to vegetative organs and N distributed to vegetative organs on the positive side of the scale, balanced by HI, NHI, NUtE, and ratios of silique husk biomass to shoot biomass on the negative side. The vectors HI, NHI had the smallest angles with NUtE in both the pot experiment and the field trial. Besides HI and NHI, vectors of seeds number per silique also had an angle with NUtE smaller than 90° in both experiments. Although angles between vectors of one-thousand seed weight and NUtE were smaller than 90°, the vector for one-thousand seed weight was short which means for this trait only a small proportion of variability was explained by principal components 1 and 2. This trait shows only a weak relationship with other traits.

## Discussion

Many researchers have reported thatcrop NUtE is effected by the environment (Han et al., 2015). For example, the NUtE of 20 winter wheat (*Triticum aestivum* L.) genotypes varied between the 2 years (Le Gouis et al., 2000). In addition, for oilseed rape, Berry et al. (2010) reported that the average NUtE under a high N supply varied between field sites. When looking for NUtE traits it is important that the phenotype is strong with a background of environmental variation (Han et al., 2015). To get a robust phenotype, ideally multiple field trials should be performed, which takes a long time and becomes expensive. Growing plants in pots or in a growth chamber should shorten the period of the experiment and reduce variation in the environment. However, in many cases the significant conclusions from the pot experiments often cannot be repeated in field trials (Mcallister et al., 2012). In the present study, the growth environment (pot experiment vs. field experiment) significantly influenced the genotype × N rate interaction effect on NUtE, agronomic and N traits (see pot v field comparisons in Supplementary Table 1, Tables 2–4). Under high N rates, the average NUtE (10.81 kg seed yield kg^−1^ shoot N uptake) of the 50 genotypes in the pot experiment was approximately one-half that in the field experiment (20.45 kg seed yield kg^−1^ shoot N uptake) (Supplementary Table 1). Furthermore, in the cultivar ranking by NUtE, most of the genotypes varied between the pot experiment and the field trial. However, although single year pot and field trials were conducted, both experiments identified some consistent genotypes as N-efficient, N-inefficient, N-responders and N-nonresponders (Figures 1C,D). The concept of N-responding and nonresponding genotypes, and efficient and inefficient genotypes is a widely-used concept in nutrient use efficiency. However, one must bear in mind that it is always a relative measurement in comparison to other genotypes within the same experiment. An efficient/responding genotype within one study can be inefficient/non-responding in another study if all other tested genotypes have a higher efficiency/response. Genotype 48 is N-responding/efficient under contrasting N supplies and genotype 44 is always an N-nonresponding/inefficient genotype when compared to genotype 48. These two genotypes can be used to identify key traits to isolate target genes and determine the physiological mechanisms that are associated with NUtE.

Although, the data of the pot and the field experiments were not correlated, principal component analysis showed similar genetic correlations between NUtE and other studied traits (Figures 2, 7). Limpens et al. (2012) conducted a meta-analysis of >200 experiments on N effects in Sphagnum, physiological responses were found to be similar in lab and field. Hohmann et al. (2016) conducted different experiments over 2 years to compare winter oilseed rape yield in multi-environment field trials. They found they were able to predict yields in the field with high accuracy from container-grown plants. Poorter et al. (2016) commented that lab-to-field extrapolations only need to deal with random variability in one year, there is a possibility that a well-designed lab experiment was better at predicting genotypic variations in the field than field data taken from a random year of field trials. They found r^2^ between lab and field phenotypic data to be larger than that between two years of field trials in meta-analysis.

For all studied N conditions, NUtE was significantly correlated with the seed number per silique in this study (Figures 2, 7), whereas no consistent correlations were found between NUtE and the other yield components under the various N supply conditions (Figures 2, 7). For the correlations between yield components and nutrient efficiency, Horst et al. (1993, 1996) contrasted two wheat cultivars with different P uptake efficiency and attributed a high grain number per ear to efficient P utilization. Svečnjak and Rengel (2006) reported that the seed number per silique was not affected by N rate among 4 spring canola cultivars differing in NUtE but that genotypic variation occurred in the seed number per silique. Mendham et al. (1981) showed a large proportion of yield could be explained by the seeds/m^2^. Berry et al. (2010) reported that among 10 oilseed rape cultivars positive association of seeds/m^2^ with yield at both low and high N conditions. Variations in seeds per silique may result from differences in sink strength, driven by variations in N remobilization and late N uptake and finally leading to NUtE differences. It is necessary to conduct further work to investigate whether the positive correlations between seed number per silique and NUtE are associated with late N uptake and N remobilization.

Chen et al. (2014) collected 488 global collections of oilseed rape to evaluate yield and other agronomic traits. They reported a decreasing effect of three yield component factors contributing to yield in the order: number of siliques per plant > seed number per silique >1000-seed weight. Diepenbrock (2000) suggested that selection for high silique number per plant in breeding is unlikely to succeed due to the low heritability in this trait. Large numbers of siliques per plant tend to be inefficient in improving yield, because many of the siliques and seeds are aborted. Both Chen et al. (2014) and Diepenbrock (2000) found a weak correlation between 1,000-seed weight and yield. Thus, seed number per silique is important for yield and N utilization in oilseed rape. In the present study, the low plant density should be noted. The influence of plant density on seed yield is mainly on the number of pods per plant. Seeds per pod and 1,000-seed weight were stable across populations (Angadi et al., 2003). Furthermore, genotypic variation in response to increasing plant density is small in oilseed rape (Diepenbrock, 2000). The observation of strong correlations between seeds per silique and NUtE in the present study may offer clues for methods to of rapidly identify breeding lines with high NUtE, especially under low planting density.

The NHI is a powerful measure of the partitioning of plant N to the seeds, reflecting the genetics of many components of N metabolism (Fageria, 2014). In this study, genotypes significantly differed in NHI under low and high N supply, and NUtE was strongly correlated with NHI (Figures 4, 7). The results agree with other reported field experiments (Fageria and Baligar, 2005; Ulas et al., 2013; Stahl et al., 2016). The genotypes with higher NUtE performed better in NHI than the genotypes with lower NUtE (Figure 6), indicating that NUtE is associated with plant N remobilization capacity, and late N uptake distribution. Leaves have been reported as the major organ for N remobilization (Gombert et al., 2010), and oilseed rape has a low N remobilization from leaves at the vegetative stage (Avice and Etienne, 2014). However, Girondé et al. (2015) reported that though enhanced N remobilization could limit N residual in fallen leaves, to improve N efficiency, the remobilized N needs to be efficiently utilized in young leaves. Furthermore, potential leaf N remobilization at the vegetative stage (Wang et al., 2016) and apparent leaf N remobilization at maturity (Ulas et al., 2013) were not correlated with genotypic variations in NUtE of oilseed rape. In the present study, at maturity, stem and stem attached leaves were harvest together. As most leaves are shed before maturity, stem attached leaves were tiny. The stem and leaf N concentration, N accumulation and ratios of stem and leaf N to shoot N were all negatively correlated with NUtE. Berry et al. (2010) and Ulas et al. (2013) also found varietal differences existed for the residual stem N in oilseed rape. It appears that residual stem N is associated with NUtE. NHI is also associated with late N uptake. Rossato et al. (2001) reported that N uptake capacity decreased at flowering to a non-significant level during pod filling, however, genotypic variations in late N uptake existed, and contributed more to NUtE differences between cultivars than stem N remobilization in selected genotypes (Berry et al., 2010; Ulas et al., 2013). Late N uptake is usually calculated as the N difference between shoot at maturity and shoot at the beginning of flowering. Dead leaves could be collected and their N concent measured. It is technically difficult to determine late N uptake. Thus, stem residual N might be a good indicator to select genotypes with high NUtE and NHI.

The HI is a measure of biological success in distributing assimilated photosynthate. Genotypes with a higher harvest index distribute more carbohydrate to the product (seeds in the case of oilseed rape; Donald and Hamblin, 1976; Li et al., 2012). It is not so surprising that HI had strong correlations with NUtE (Figures 4, 7). Significant correlations between NUtE and HI were observed in barley (Bingham et al., 2012), in wheat (Ortiz-Monasterio et al., 2001) and in Brussels sprouts (Fiedler and Stützel, 2012). Diepenbrock (2000) reviewed HI to be a limiting yield parameter in oilseed rape, with higher HI leading to higher grain yield. Among both selected lines (Berry et al., 2010) and DH population (Miro, 2010; Nyikako et al., 2014) NUtE is associated with HI in oilseed rape. In the present study, NUtE was negatively correlated with biomass distribution to stem under two contrasting N treatments while only at low N supplies, negative correlation coefficients were observed between NUtE and biomass distribution to silique husk. Compared with wheat, oilseed rape had a lower HI and a lower NHI (Dreccer et al., 2000). Therefore, these traits may be effective in selecting oilseed rape genotypes with nitrogen efficient utilization (Cregan and Van Berkum, 1984; Ortiz-Monasterio et al., 2001).

Root samples, which are difficult to collect in the field, were studied only in the pot experiment in the present study. The limited space of the pot for root growth should be noted. Although the root development may be limited, under a high N rate NUtE was negatively correlated with root biomass, and the N-responder genotypes had a lower root biomass than did the N-nonresponder genotypes. The root-to-plant ratio for biomass is a measure of carbohydrate partitioning between the shoot and the root. NUtE was negatively correlated with this ratio in oilseed rape under high N supply, and the ratios of the N-responder genotypes were significantly lower than those of the N-nonresponder genotypes. In addition, Svečnjak and Rengel (2006) reported that a canola cultivar with high N efficiency at grain harvest had a lower root-to-shoot ratio for biomass than did another cultivar with low N efficiency. In contrast, previous pot studies (Cao et al., 2010) showed that oilseed rape genotypes with high N uptake efficiency (plant N uptake per N applied) had significantly higher root biomasses, and significant larger ratios of root biomass to whole plant biomass than did those with low N uptake efficiency. These results suggest that the root plays different roles in N utilization efficiency and N uptake efficiency. The root-to-plant ratio for N accumulation is an indicator of the biological processes occurring during N allocation. Under the high-N supply, NUtE was significantly and negatively correlated with the root-to-plant ratio for N accumulation, and the N-responder genotypes had lower ratios of root N to plant N than did the N-nonresponder genotypes. Thus, the genotypes with high NUtE tended to distribute more photosynthate and N to the shoot than did the genotypes with low NUtE.

## Conclusions

Improving NUtE of oilseed rape is of great importance for growing the crop on low N soils without decreasing seed yield. This goal can be facilitated by using simplified growth approaches that allow the use of molecular tools to determine the genetic background of a phenotype. However, identifying robust traits across simplified and complex environments to improve NUtE in oilseed rape is the first step in this research.

In the present study, the NUtE, agronomic and morphological traits of 50 oilseed rape genotypes were examined and compared at contrasting N conditions across controlled (pot) and complex (field) environments. Despite only 1-year of pot and field experiments, seeds number per silique, harvest index and N harvest index were significantly correlated with NUtE under contrasting N conditions in all environments. Seeds number per silique may be a good indicator in rapidly selecting N efficient genotypes. These analyses suggested that this oilseed rape population which included 50 oilseed rape genotypes with various genetic backgrounds, had remarkably similar underlying sub-traits of NUtE and some genotypes performed consistently across these environments. Further field trials are necessary to confirm the importance of these traits in varied environments.

## Author contributions

HH analyzed the data and wrote the manuscript; RY took part in designing the field experiment, conducted it, acquired the data and participated in drafting the work; YL and LC took part in designing the pot experiment, conducted it, acquired the data and participated in drafting the work; AM conducted the field experiment and acquired the data; XW and BC took part in designing the field and pot experiment and drafting the work, and also provided the 50 oilseed rape genotypes; YG designed the pot and field experiments, conducted it, acquired and analyzed the data. He also drafted the work and revised the manuscript critically. HT edited the manuscript. HH, RY, YL, LC, AM, XW, BC, HT, and YG they all approved the manuscript to be published and agreed to be accountable for all aspects of the work in ensuring the questions related to the accuracy or integrity of any part of the work are appropriately investigated and resolved.

### Conflict of interest statement

The authors declare that the research was conducted in the absence of any commercial or financial relationships that could be construed as a potential conflict of interest.

## Supplementary material

Supplementary Figure 1 Plant section biomass and whole-plant biomass of the N-responder and N-nonresponder genotypes under high N rates in a pot experiment **(A)** and a field experiment **(C)**, and the biomass of the N-efficient and N-inefficient genotypes under low N rates in the pot experiment **(B)** and the field experiment **(D)**. Values represent means ± SD. Bars with different letters indicate significant differences between the different NUtE genotypes at *p* < 0.05. SL, stem and leaf; SH, silique husk.

Supplementary Figure 2 N concentration of different plant sections under high N rates in the pot experiment **(A)** and the field experiment **(C)** and N concentrations under low N rates in the pot experiment **(B)** and the field experiment **(D)**. Values represent means ± SD. Bars with different letters indicate significant differences at *p* < 0.05.

Supplementary Figure 3 N accumulation in different plant sections under high N rates in the pot experiment **(A)** and field experiment **(C)** and N accumulation under low N rates in the pot experiment **(B)** and field experiment **(D)**. Values represent means ± SD. Bars with different letters indicate significant differences at *p* < 0.05. SL, stem and leaf; SH, silique husk.

Supplementary Table 1 NUtE of 50 oilseed rape genotypes in the pot experiment and in the field trial.
