# Supplementary figures and images for: Genotypic Variation in Nitrogen Utilization Efficiency of Oilseed Rape (Brassica napus) Under Contrasting N Supply in Pot and Field Experiments
Source: Front Plant Sci. 2017 Oct 27;8:1825. doi: 10.3389/fpls.2017.01825 (PMC5664426; doi:10.3389/fpls.2017.01825)

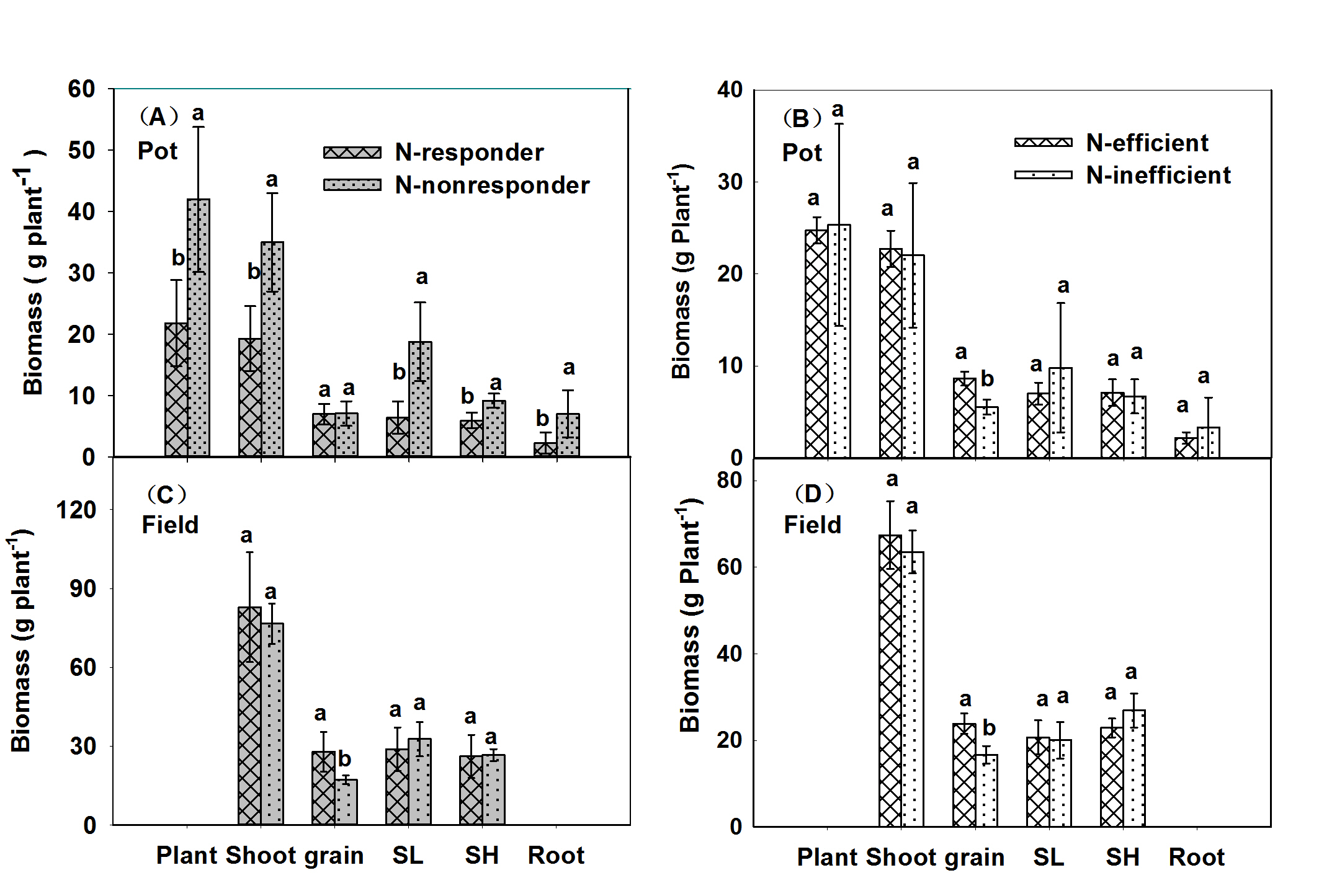

Supplement: Supplementary Figure 1 — Plant section biomass and whole-plant biomass of the N-responder and N-nonresponder genotypes under high N rates in a pot experiment (A) and a field experiment (C), and the biomass of the N-efficient and N-inefficient genotypes under low N rates in the pot experiment (B) and the field experiment (D). Values represent means ± SD. Bars with different letters indicate significant differences between the different NUtE genotypes at p < 0.05. SL, stem and leaf; SH, silique husk. [file Image1.JPEG]

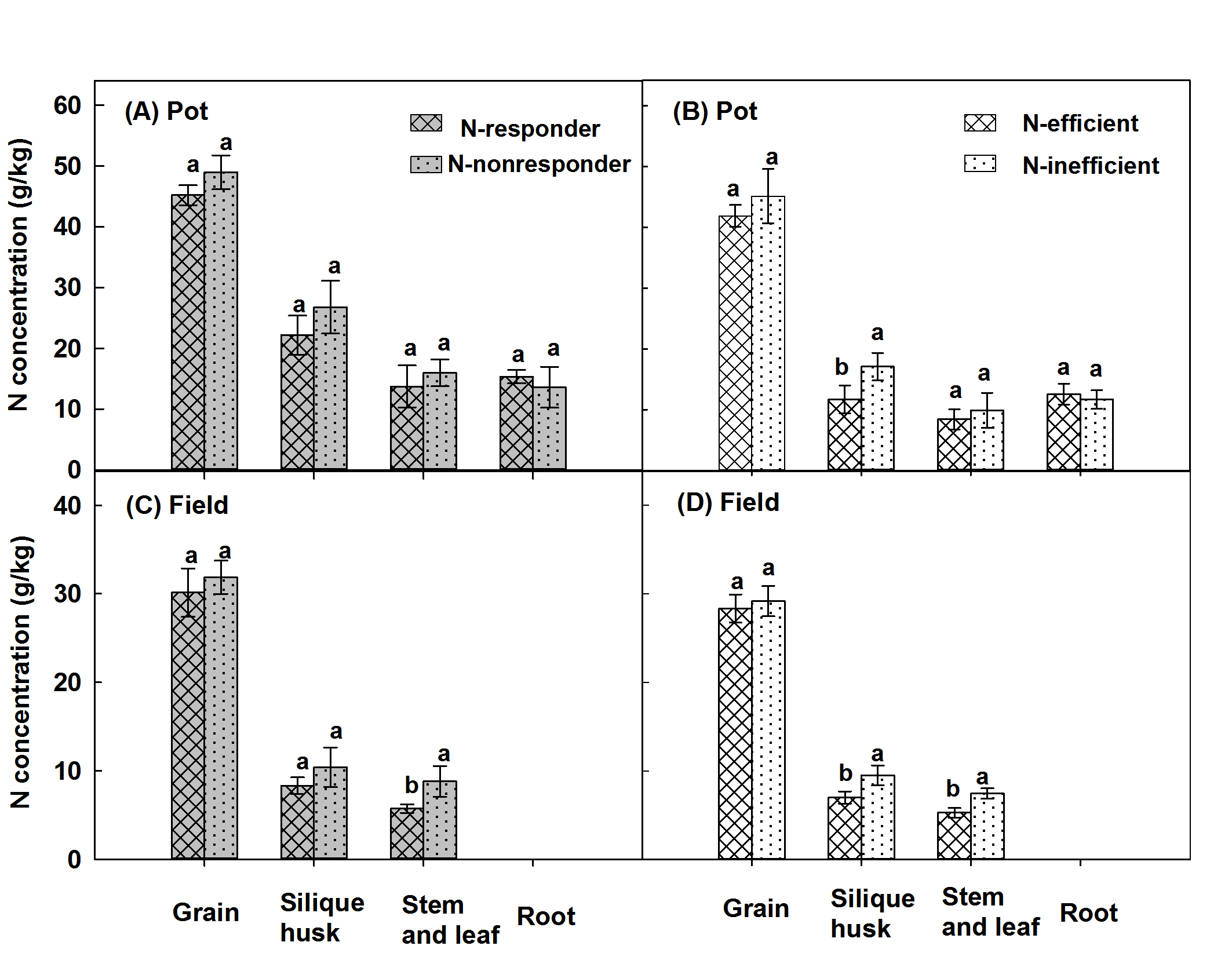

Supplement: Supplementary Figure 2 — N concentration of different plant sections under high N rates in the pot experiment (A) and the field experiment (C) and N concentrations under low N rates in the pot experiment (B) and the field experiment (D). Values represent means ± SD. Bars with different letters indicate significant differences at p < 0.05. [file Image2.JPEG]

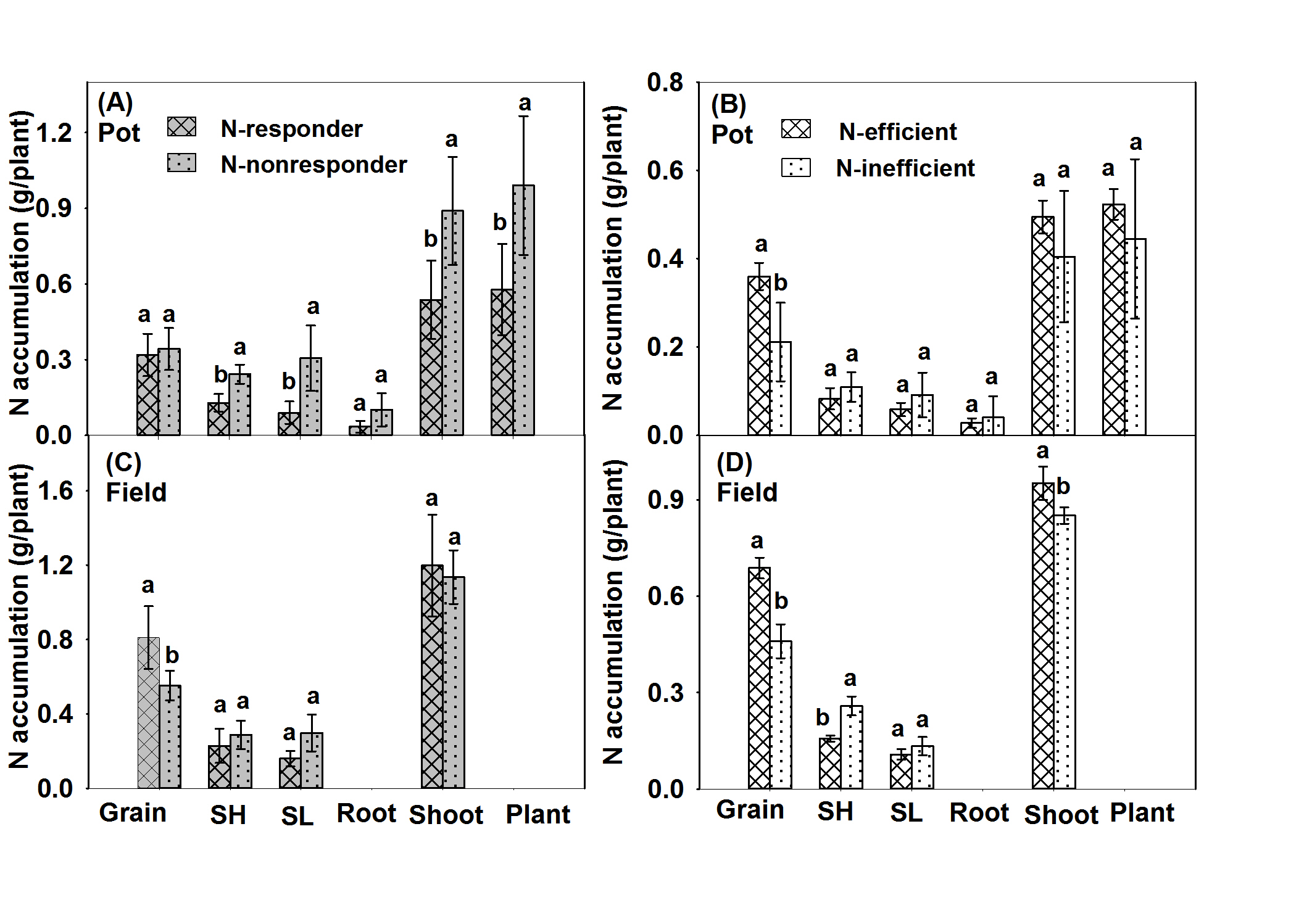

Supplement: Supplementary Figure 3 — N accumulation in different plant sections under high N rates in the pot experiment (A) and field experiment (C) and N accumulation under low N rates in the pot experiment (B) and field experiment (D). Values represent means ± SD. Bars with different letters indicate significant differences at p < 0.05. SL, stem and leaf; SH, silique husk. [file Image3.JPEG]
