# Supplementary material for: Genotypic Variation in Nitrogen Utilization Efficiency of Oilseed Rape (Brassica napus) Under Contrasting N Supply in Pot and Field Experiments
Source: Front Plant Sci. 2017 Oct 27;8:1825. doi: 10.3389/fpls.2017.01825 (PMC5664426; doi:10.3389/fpls.2017.01825)
Supplement: Supplementary Table 1 — NUtE of 50 oilseed rape genotypes in the pot experiment and in the field trial. [file Table1.DOCX]

Supplementary Material

**Genotypic variation in nitrogen utilization efficiency of oilseed rape (*Brassica napus*) under contrasting N supply in pot and field experiments**

**Huiying He^1, 3^, Rui Yang^1^, Yajun Li^1^, Lanqin Cao^1^, Xiaoming Wu^2^, Biyun Chen^2^, Hui Tian^1^, Yajun Gao^1, 3^***

^1^College of Natural Resource and Environment, Northwest A&F University, Yangling, Shaanxi, China

^2^Institute of Oil Crop Research, Chinese Academy of Agricultural Sciences, Wuhan, China

^3^Key Laboratory of Plant Nutrition and the Agri-environment in Northwest China, Ministry of Agriculture, Yangling, Shaanxi*** Correspondence: Yajun Gao, College of Natural Resource and Environment, Northwest A&F University, Yangling, Shaanxi, 712100, China**

[yajungao@nwsuaf.edu.cn](mailto:yajungao@nwsuaf.edu.cn)

Table S1 NUtE of 50 oilseed rape genotypes in the pot experiment and in the field trial

| High N rates | | | | |  | Low N rates | | | | |
| --- | --- | --- | --- | --- | --- | --- | --- | --- | --- | --- |
| genotypes | NUtE  in the pot experiment |  | genotypes | NUtE  in the field trial |  | genotypes | NUtE  in the pot experiment |  | genotypes | NUtE  in the field trial |
| 34 | 14.83±0.62 |  | 17 | 31.46±11.35 |  | 23 | 21.20±2.42 |  | 40 | 29.33±4.56 |
| 40 | 14.35±0.99 |  | 2 | 25.95±3.71 |  | 34 | 20.44±0.93 |  | 13 | 29.22±4.42 |
| 49 | 13.97±1.86 |  | 9 | 25.57±3.33 |  | 25 | 18.85±1.95 |  | 16 | 28.57±5.14 |
| 37 | 13.94±1.86 |  | 16 | 24.42±8.20 |  | 21 | 18.78±1.15 |  | 24 | 26.69±4.52 |
| 48 | 13.51±1.07 |  | 20 | 24.16±3.38 |  | 14 | 18.64±0.47 |  | 10 | 26.59±0.45 |
| 7 | 13.48±2.28 |  | 36 | 23.69±4.66 |  | 27 | 18.23±3.87 |  | 48 | 26.26±2.72 |
| 18 | 13.47±2.86 |  | 13 | 23.55±5.76 |  | 26 | 18.06±1.59 |  | 2 | 26.26±2.10 |
| 31 | 12.89±1.10 |  | 48 | 23.41±1.47 |  | 37 | 18.05±3.90 |  | 41 | 25.88±5.69 |
| 35 | 12.70±3.11 |  | 40 | 23.40±2.88 |  | 48 | 17.91±1.33 |  | 45 | 25.30±0.91 |
| 28 | 12.42±0.60 |  | 1 | 22.98±3.06 |  | 42 | 17.81±0.26 |  | 42 | 24.62±2.97 |
| 8 | 12.32±1.28 |  | 28 | 22.79±3.56 |  | 31 | 17.74±0.78 |  | 4 | 24.61±2.67 |
| 9 | 11.95±1.47 |  | 10 | 22.67±2.33 |  | 29 | 17.52±2.01 |  | 20 | 24.28±2.66 |
| 30 | 11.95±1.47 |  | 5 | 22.41±1.71 |  | 45 | 17.49±5.95 |  | 43 | 24.25±3.25 |
| 29 | 11.84±1.63 |  | 7 | 22.27±2.92 |  | 32 | 17.36±1.62 |  | 30 | 24.19±4.01 |
| 23 | 11.77±1.90 |  | 6 | 22.21±2.21 |  | 20 | 17.24±0.26 |  | 17 | 24.07±1.25 |
| 1 | 11.70±1.48 |  | 37 | 22.04±1.98 |  | 49 | 17.23±1.28 |  | 5 | 24.01±5.17 |
| 2 | 11.57±1.72 |  | 47 | 21.60±3.04 |  | 24 | 17.21±2.38 |  | 46 | 23.63±4.55 |
| 46 | 11.49±0.10 |  | 4 | 21.46±4.01 |  | 22 | 16.64±1.06 |  | 21 | 23.36±7.44 |
| 15 | 11.46±1.25 |  | 3 | 21.35±4.10 |  | 15 | 16.60±1.01 |  | 33 | 23.34±2.92 |
| 45 | 11.44±2.00 |  | 31 | 21.04±2.77 |  | 28 | 16.44±0.83 |  | 47 | 23.31±3.75 |
| 32 | 11.30±2.66 |  | 14 | 21.03±4.67 |  | 46 | 16.33±1.13 |  | 9 | 23.14±2.07 |
| 3 | 11.03±0.30 |  | 42 | 20.86±2.30 |  | 5 | 16.12±3.90 |  | 34 | 22.68±5.76 |
| 10 | 11.02±1.19 |  | 24 | 20.75±7.50 |  | 30 | 15.85±2.32 |  | 31 | 22.56±5.11 |
| 36 | 10.93±1.87 |  | 8 | 20.68±9.84 |  | 12 | 15.66±0.66 |  | 28 | 22.54±4.73 |
| 16 | 10.83±1.52 |  | 41 | 20.31±4.14 |  | 7 | 15.61±1.60 |  | 11 | 22.50±4.83 |
| 38 | 10.81±0.80 |  | 43 | 20.21±4.15 |  | 50 | 15.55±2.06 |  | 32 | 22.37±3.73 |
| 13 | 10.63±1.02 |  | 46 | 19.85±3.40 |  | 16 | 15.49±0.83 |  | 1 | 22.14±5.52 |
| 4 | 10.55±2.53 |  | 29 | 19.83±5.90 |  | 17 | 15.47±1.39 |  | 29 | 22.12±8.57 |
| 20 | 10.53±3.74 |  | 38 | 19.81±3.43 |  | 19 | 15.00±3.08 |  | 38 | 21.75±3.81 |
| 6 | 10.33±1.23 |  | 19 | 19.69±3.84 |  | 35 | 14.88±1.71 |  | 36 | 21.63±6.40 |
| 39 | 10.17±2.24 |  | 12 | 19.26±5.43 |  | 36 | 14.68±1.09 |  | 37 | 21.55±7.07 |
| 42 | 10.16±1.83 |  | 34 | 19.25±7.89 |  | 6 | 14.67±2.26 |  | 3 | 21.39±1.80 |
| 5 | 10.12±2.52 |  | 21 | 19.02±2.15 |  | 9 | 14.34±1.10 |  | 50 | 21.29±2.91 |
| 14 | 10.09±0.64 |  | 11 | 18.89±4.76 |  | 38 | 14.16±0.64 |  | 7 | 21.15±6.64 |
| 24 | 9.93±3.09 |  | 39 | 18.89±5.53 |  | 43 | 14.00±2.08 |  | 22 | 21.13±1.37 |
| 17 | 9.58±1.77 |  | 32 | 18.72±4.03 |  | 1 | 13.06±1.16 |  | 15 | 20.99±6.61 |
| 21 | 9.55±4.90 |  | 23 | 18.63±3.00 |  | 40 | 12.88±1.28 |  | 12 | 20.90±5.45 |
| 12 | 9.45±0.98 |  | 49 | 18.53±10.71 |  | 3 | 12.73±2.03 |  | 25 | 20.76±5.84 |
| 19 | 9.36±1.19 |  | 45 | 18.26±5.45 |  | 39 | 12.73±3.10 |  | 6 | 20.57±5.81 |
| 26 | 9.28±0.85 |  | 35 | 18.14±0.79 |  | 10 | 12.61±0.42 |  | 19 | 20.28±5.78 |
| 22 | 9.23±0.97 |  | 33 | 17.96±5.47 |  | 41 | 12.46±0.80 |  | 18 | 20.01±1.71 |
| 11 | 9.21±0.93 |  | 15 | 17.86±5.32 |  | 2 | 12.14±5.53 |  | 8 | 19.62±8.51 |
| 43 | 8.88±2.44 |  | 30 | 17.51±4.33 |  | 8 | 11.86±3.66 |  | 35 | 19.05±2.44 |
| 33 | 8.84±2.36 |  | 25 | 17.40±7.22 |  | 44 | 11.75±3.86 |  | 26 | 19.04±7.27 |
| 41 | 8.44±0.82 |  | 22 | 17.33±6.50 |  | 11 | 11.19±3.22 |  | 39 | 18.98±8.16 |
| 27 | 8.05±0.42 |  | 50 | 16.94±1.47 |  | 47 | 11.15±1.97 |  | 49 | 17.96±4.61 |
| 47 | 7.78±1.55 |  | 18 | 16.39±6.91 |  | 18 | 11.06±3.76 |  | 23 | 17.95±3.85 |
| 50 | 7.57±3.19 |  | 27 | 16.30±11.12 |  | 4 | 11.00±1.81 |  | 44 | 17.60±6.09 |
| 44 | 6.98±0.65 |  | 26 | 15.21±8.56 |  | 13 | 9.52±0.40 |  | 14 | 17.38±4.22 |
| 25 | 6.65±0.87 |  | 44 | 12.35±4.82 |  | 33 | 7.59±0.73 |  | 27 | 17.16±4.67 |
